# High throughput analysis of contaminants of emerging concern in complex matrices

**DOI:** 10.1016/j.mex.2026.104201

**Published:** 2026-10-10

**Authors:** Tsz Yung (Patrick) Wong, Bent Speksnijder, Gayani R. Kapukotuwa, Oksana Golovko

**Affiliations:** a Department of Aquatic Sciences and Assessment, Swedish University of Agricultural Sciences (SLU), Uppsala, SE-75007, Sweden; b University of South Bohemia in České Budějovice, Faculty of Fisheries and Protection of Water, South Bohemian Research Center of Aquaculture and Biodiversity of Hydrocenoses, Zátiší 728/II, Vodňany, CZ-389 25, Czech Republic; c Department of Energy and Technology, Swedish University of Agricultural Sciences (SLU), Box 7032, Uppsala, SE-75007, Sweden

**Keywords:** PFAS, Pharmaceuticals, Industrial chemicals, Online SPE, UPLC-MS/MS

## Abstract

Contaminants of emerging concern (CECs) occur across diverse aquatic matrices, but their reliable quantification remains challenging because of large differences in matrix composition. This study developed and validated a rapid online solid-phase extraction coupled with ultra-high-performance liquid chromatography–tandem mass spectrometry (online-SPE-UPLC-MS/MS) method for the simultaneous determination of 46 pharmaceuticals, industrial chemicals, pesticides, and per- and polyfluoroalkyl substances (PFAS). Method performance was evaluated in six matrices: tap water, river water, wastewater influent and effluent, and untreated and treated landfill leachate. The method was validated by evaluating compound- and matrix-dependent limits of quantification (LOQs), matrix effects and recoveries. Pronounced matrix-induced ion suppression was observed before internal-standard correction, particularly in wastewater influent and untreated landfill leachate. Most analytes showed recoveries within 50–150% and precision below 30% relative standard deviation. In addition, stability was tested, which was highly matrix and storage dependent, with freezing generally providing improved preservation. Validation was included in method application for untreated and treated leachates from five Swedish landfills.

A multi-class analytical method was developed and evaluated in six matrices

The method was validated using matrix-specific assessment of sensitivity, accuracy, precision, recovery, matrix effects, and stability during storage.

The method was applied to untreated and treated leachates from five Swedish landfills.

## Graphical abstract

**Figure:**
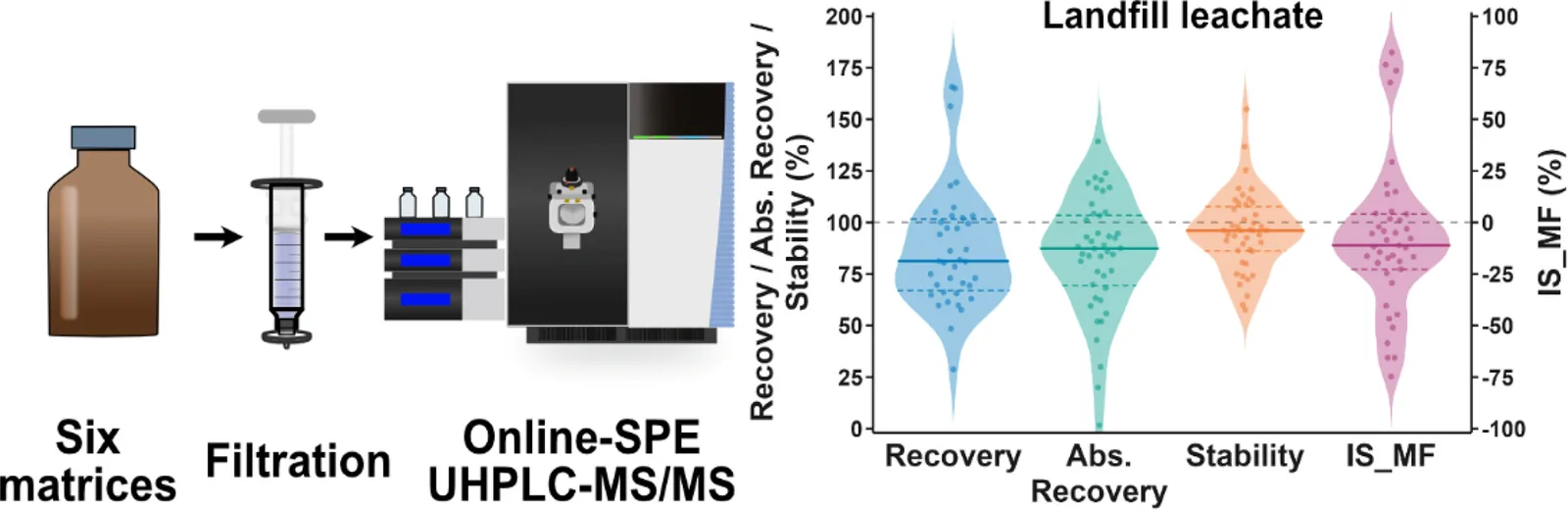

## Specifications table

**Table:** 

**Subject area**	Environmental Science
**More specific subject area**	*Environmental Chemistry - Organic Contaminants*
**Name of your method**	*PFAS, pharmaceuticals, and industrial chemicals in wastewater, untreated and treated landfill leachate, river water, and tap water.*
**Name and reference of original method**	*This analytical method is based on the method described by* [1] *Evaluation of targeted and high-resolution mass spectrometry methods for environmental monitoring of pharmaceuticals,*https://doi.org/10.1016/j.mex.2025.103666
**Resource availability**	*Not applicable*

## Background

Aquatic environments receive a diverse mixture of contaminants originating from industrial, municipal and consumer activities. Contaminants of emerging concern (CECs), including industrial chemicals, pharmaceuticals and personal care products, and per- and polyfluoroalkyl substances (PFAS), are of particular concern because many of these compounds are persistent, biologically active, or have been associated with adverse effects on aquatic ecosystems [2]. Wastewater treatment plants represent one of the most common pathways through which such CECs enter receiving waters [3]. Although treatment processes can reduce the concentrations of some substances, many CECs are only partially removed or transformed into products that may stay in the environment [4]. Landfill leachate is another important source of complex CECs mixture [5]. Recent non-target screening studies have revealed the presence of numerous CECs in both treated and untreated landfill leachate [6]. These findings demonstrate the chemical complexity of the leachate and highlight its potential contribution to contaminant loading in wastewater treatment systems and aquatic environment.

Despite advances in analytical instrumentation, quantitative methods capable of simultaneously determining such a diverse range of contaminants with different physicochemical properties remain limited. Conventional analytical approaches often require compound-specific extraction procedure, extensive sample preparation or multiple instrumental methods [7]. These requirements increase analytical time, cost and workload, thereby restricting the number of samples that can be included in the environmental monitoring programs. Complex matrices such as wastewater and landfill leachate also present substantial analytical challenges because high concentrations of dissolved organic matter, salts and other matrix components may interfere with contaminant detection and quantification. A simple, reliable and high-throughput analytical methodology is therefore needed to quantify diverse contaminants across multiple environmental water matrices while minimizing sample preparation and analytical workload [1]. Such a method would support large-scale monitoring, improve understanding of contaminant occurrence and transport, and provide data for environmental risk assessment and management.

In this study, six water matrices representing different stages of contaminant release, treatment, environmental exposure, and human relevance were investigated. These include wastewater influent (WWI) and untreated landfill leachate (LU), which are characterized by high contaminant loads. Wastewater effluent (WWE), treated landfill leachate (LT), and river water (RW), which represent potential pathways to the aquatic environment. Tap water (TW) was included because of its direct relevance to human exposure and public health. This study aimed to develop and evaluate a broadly applicable quantitative method for determining contaminants across these chemically diverse matrices.

## Method details

### Chemicals, reagents and materials

LC/MS-grade methanol (MeOH) and acetonitrile were obtained from Sigma Aldrich (Sweden). Formic acid (LC/MS-grade) was used for mobile-phase acidification and was purchased from Fisher Chemical (Sweden). Milli-Q water was obtained from a Milli-Q IQ 7000 and filtered through an LC-pak for the mobile phases and through a 0.22 µm Millipack membrane filter (Merck KGaA, Darmstadt, Germany) for other uses.

A total of 46 native compounds and 16 internal standards (ISs) were used. Stock spiking solutions of native compounds and ISs were prepared in MeOH at concentrations of 1 μg/mL and stored at –20°C until use. Detailed information on chemicals, reagents, and materials is provided in the Supplementary Information (SI), Table S1.

### Experimental setup

The method was adopted from Golovko et al. [1] who established an online-SPE method for the measurement of pharmaceuticals in Tap Water (TW), River Water (RW), Wastewater Influent (WWI) and Wastewater Effluent (WWE). In this study, several changes have been implemented, including the use of a BEH C18 column, the measurement of several industrial chemicals and PFAS, and the analysis of treated and untreated landfill leachate (LT and LU, respectively).

### Sample collection

Prior to sample collection, HDPE bottles were washed with 3x MilliQ and 3x ethanol and dried for 24 hours under the fume hood. Samples were collected and stored at +4°C until sample preparation within 24 hours. WWI and WWE were collected from municipal wastewater treatment plant (WWTP) in Uppsala (200 000 population equivalent). Treatment at the WWTP includes several stages, including anaerobic digestion and flocculation using FeCl_3_ [8], before it is released into the river Fyris. RW was collected downstream of the local WWTP in the river Fyris.

LT and LU were collected at the municipal landfill. This landfill contains a mixture of municipal waste, sludge storage and construction waste, among others. Treatment includes aeration, a biofilm reactor, sedimentations ponds followed by a fibre disc filtration for phosphorous removal [9].

For the method application section, leachate samples were collected from five landfills in Sweden in June and July of 2023. Detailed information regarding the landfill, waste type, and leachate treatment was reported previously [6].

### Liquid chromatography

The Samples were analysed using an Ultimate 3000 Ultra-High-Performance Liquid Chromatography (UPLC) system (Thermo Scientific, Waltham, MA, USA) connected to a CTC PAL autosampler (Thermo Scientific, Waltham, MA, USA) with a cooling unit set at 10°C.

The online SPE extraction column was a Hypersil GOLD™ aQ C18 Reversed Phase HPLC Column (12 μm, 2.1 mm x 20 mm), Thermo Scientific™. The analytical column was an ACQUITY UPLC BEH C18 Column (1.7 µm, 2.1 mm x 50 mm), Waters. The mobile phases consisted of MilliQ water with 0.1% formic acid (A) and acetonitrile with 0.1% formic acid (B). The total run time was 14 min, and the gradient details can be found in the SI, Table S2. In short: 1 mL of the sample was injected into a 1 mL sample loop. This was then transported to the extraction column for 1.25 min. Afterwards, the analytical column released the captured compound using gradient starting at 5% (B) up towards 100% (B) at 7.5 min, this was maintained till 11 min, before the column was re-equilibrated to their started positions. Prior to analysis of each matrix, the system was rinsed with four injections of Milli-Q water. System backpressure was monitored throughout each analytical sequence to ensure stable LC performance.

### Tandem mass spectrometry

A triple quadrupole mass spectrometer (QUANTIVA, Thermo Scientific, Waltham, MA, USA) equipped with a HESI spray was coupled to the UPLC system. The positive spray voltage was set to 3500 V, the negative to 2500 V and the vaporizer temperature was set to 400°C. Other details can be found in the SI, Table S3.

The system was using targeted MS/MS analysis using Multiple Reaction Monitoring (MRM) to analyse the different compounds, and the data was processed using TraceFinder™ 4.1. Details on the different transitions can be found in the MRM table in the SI, Table S4.

## Method validation

### Sample preparation for method validation

The performance of the method was evaluated by comparing limits of quantification (LOQ), accuracy, precision, recovery, matrix effects, and stability of the compounds in each of the six matrices.

To evaluate method recovery, absolute recovery, and matrix effects, each matrix was divided into eight aliquots (50 mL) in separate HDPE bottles. Fortified samples were prepared by spiking six aliquots with native standards prior to filtration with concentration levels of (1, 5, 10, 50, 100, and 500 ng/L). One unspiked aliquot served as a both a control, and a matrix matched standard (MST) which was spiked with a native standard at 500 ng/L after filtration. All samples (n=5 per matrix) were filtered through a regenerated cellulose Syringe Filter (0,45 µm, Sartorius Minisart™). Afterwards, samples were spiked using IS mixes at 50 ng/L. Surrogate internal standards were selected considering, as far as possible, chemical/structural similarity, chromatographic retention time, ionization polarity, and comparable response to matrix effects, see Table S5.

The LOQ was determined based on accuracy and precision at the lowest concentration level, in accordance with ICH Q2(R2) guidelines [10]. An external calibration curve consisting of seven points (0.5 to 500 ng/L in MilliQ water) was utilized. Fresh calibration series were prepared daily and analysed in duplicate. The LOQ was defined as the lowest level calibration level that met the acceptance criteria for precision (RSD<30%) and accuracy (back calculated recovery between 50–150%) across a total of 11 replicate injections. Matrix-dependent LOQ (mLOQ) was defined as LOQ divided by matrix effect.

The matrix effect was used to show the suppression or enhancement in different matrices and was calculated using the following equation:

(1) $$Matrix\;effect\;\left(\%\right)=\left(\frac{Slope_{\left(Matrix,\;native\right)}\;}{Slope_{\left(Solvent,\;native\right)}}\right)\times \;100\%$$

Where:

$Slope_{\left(Matrix,\;native\right)}$ = slope is based on the native compounds’ peak area *vs.* spiked concentration, obtained from MST relative to the unspiked sample.

$Slope_{\left(Solvent,\;native\right)}$ = slope is based on the native compounds’ peak area *vs.* concentration, obtained from the calibration curve.

IS-Normalized Matrix Factor (IS_MF) was used to show the relative suppression or enhancement after correction with the IS and was calculated using the following equation:

(2) $$IS\_MF\;\left(\%\right)=\left(\frac{Slope_{\left(Matrix\right)}\;}{Slope_{\left(Solvent\right)}}\right)\times \;100\%$$

Where:

$Slope_{\left(Matrix\right)}$ = slope is based on the peak area ratio (native/IS) *vs.* spiked concentration, obtained from MST relative to the unspiked sample.

$Slope_{\left(Solvent\right)}$ = slope is based on the peak area ratio (native/IS) *vs.* concentration, obtained from the calibration curve.

The concentrations were calculated using the following formula:

(3) $$C=\;\left(\frac{AR_{Native/IS}\;}{Slope_{\left(Matrix\right)}}\right)*C_{IS}-C_{Blank}$$

Where:

$Slope_{\left(Matrix\right)}$ = slope is based on the peak area ratio (native/IS) *vs.* spiked concentration, obtained from MST relative to the unspiked sample.

$AR_{Native/IS}$ = The area ratio between the native compound and its corresponding IS.

$C_{IS}$ = The concentration of the IS in the sample

$C_{Blank}$ = The concentration in the blank samples

The recovery was calculated separately for each spiking level (1, 5, 10, 50, 100 and 500 ng/L) and comparing it to the unspiked matrix (0 ng/L), it has to be noted that concentrations below LOQ were treated as 0:

(4) $$Recovery=\;\frac{{\bar{C}}_{Spiked}-{\bar{C}}_{Unspiked}}{C_{theoretical}}\;\times \;100\%$$

Where:

${\bar{C}}_{Spiked}$ = The mean concentration in the spiked matrix samples

${\bar{C}}_{Unspiked}$ = The mean concentration in the unspiked matrix samples

$C_{theoretical}$ = The spiked theoretical concentration

The precision was calculated based on the RSD calculated from the concentrations of the quintuplets at the 6 spiked and 1 unspiked sample:

(5) $$RSD=\;\frac{SD_{C}}{\bar{C}}\;\times \;100\%$$

Where:

$SD_{C}$ = Standard deviation of the concentration.

$\bar{C}$ = Concentration mean across the quintuplets.

Absolute recovery, reflecting losses associated with sample preparation, was assessed by comparing analyte peak areas in MST and fortified samples across the different water matrices:

(6) $$Absolute\;Recovery=\left(\frac{Area_{Fortified}-Area_{Unspike}}{Area_{MST}-Area_{Unspike}}\right)\times \;100\%$$

Where:

$Area_{Frotifieds}$ = Peak area native compounds of fortified samples

$Area_{Unspike}$ = Peak area native compounds of unspiked sample

$Area_{MST}$ = Peak area native compounds of MST

To assess the stability of the measured compounds, two HDPE bottle of each matrix were spiked at 500 ng/L. Samples were consequently stored in the fridge for 1 week (1 W) at +4°C or 4 weeks (4 W) in the freezer at -22°C. The stability of studied compounds was calculated using the following equation:

(7) $$Stability=\;\left(\frac{Area\;Ratio_{\left(t\right)}}{Area\;Ratio_{\left(ref\right)}}\right)\times \;100\%$$

Where:

$Area\;Ratio_{\left(t\right)}$ = Peak area ratio between native compounds and IS at storage time of 4 W in freezer or 1 W fridge condition

$Area\;Ratio_{\left(ref\right)}$ = Peak area ratio between native compounds and IS at storage time of 0 as reference.

### Matrix specific recoveries

The matrix specific recoveries were evaluated at six spiking levels in six matrices (Fig. 1). Recovery was less variable in the cleaner matrices (TW, RW) across all spiking levels, whereas the chemically complex, high-organic-load matrices (WWI, LU) showed greater scatter and a tendency toward recovery losses at higher spiked levels. Recovery for most compounds fell within the 50–150% acceptance window, though a subset of compounds exceeded these bounds at the lower spiking levels, reflecting the greater relative uncertainty near the LOQ. Notably, the spread in recovery narrowed with increasing spike concentration, indicating improved method precision at higher, more readily quantifiable concentrations. It must be highlighted, however, that calculations could result in overestimated recovery, especially at lower concentrations, due to the unspiked level being below the LOQ. Furthermore, several compounds had a surrogate IS, which may be suppressed stronger or weaker than the native compound, resulting in larger deviations in the recovery.

**Fig. 1:**
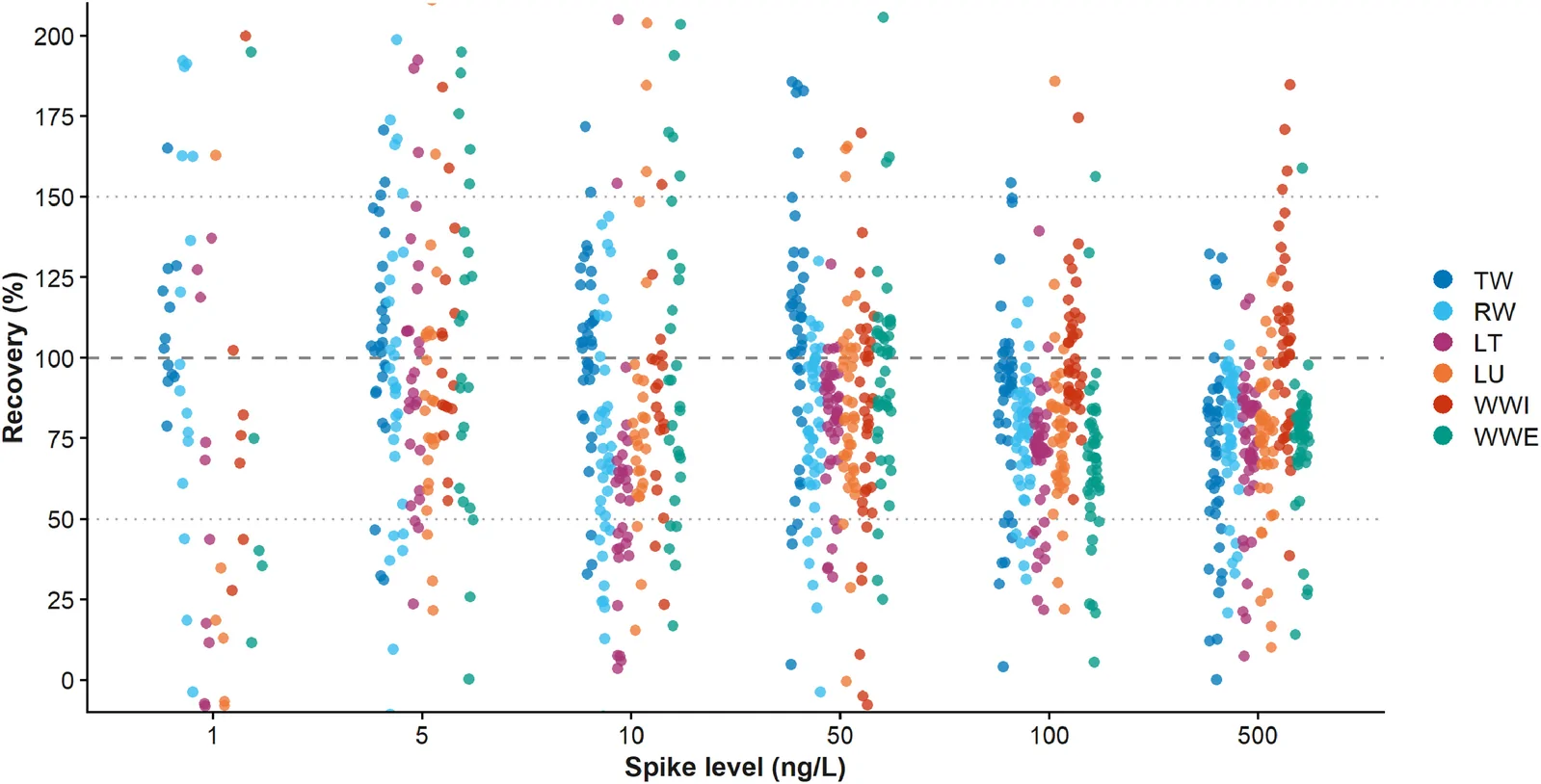
Matrix and concentration-dependent recovery of the analysed compound. Each point represents the mean percent recovery of an individual compound (n=5) at a given spiking level (1, 5, 10, 50, 100, and 500 ng/L) in six water matrices: tap water (TW), river water (RW), treated landfill leachate (LT), untreated landfill leachate (LU), wastewater influent (WWI), and wastewater effluent (WWE). The dashed line indicates 100% recovery; dotted lines indicate the acceptable recovery range (50–150%).

Fig. 2 highlights the precision of the method with most compounds falling within the acceptable RSD of <30%, with most falling even below <20%. Some exceptions were observed with especially the compound Triethyl phosphate (TEP) showing lower precisions in most matrices. In RW more compounds showed lower precision mainly being; Tributyl phosphate (TBP), Metoprolol and Atenolol acid. Nevertheless, these results demonstrate that the method maintains acceptable accuracy and precision across a wide range of matrix complexities and concentrations relevant to the full contaminant release-to-exposure pathway investigated in this study.

**Fig. 2:**
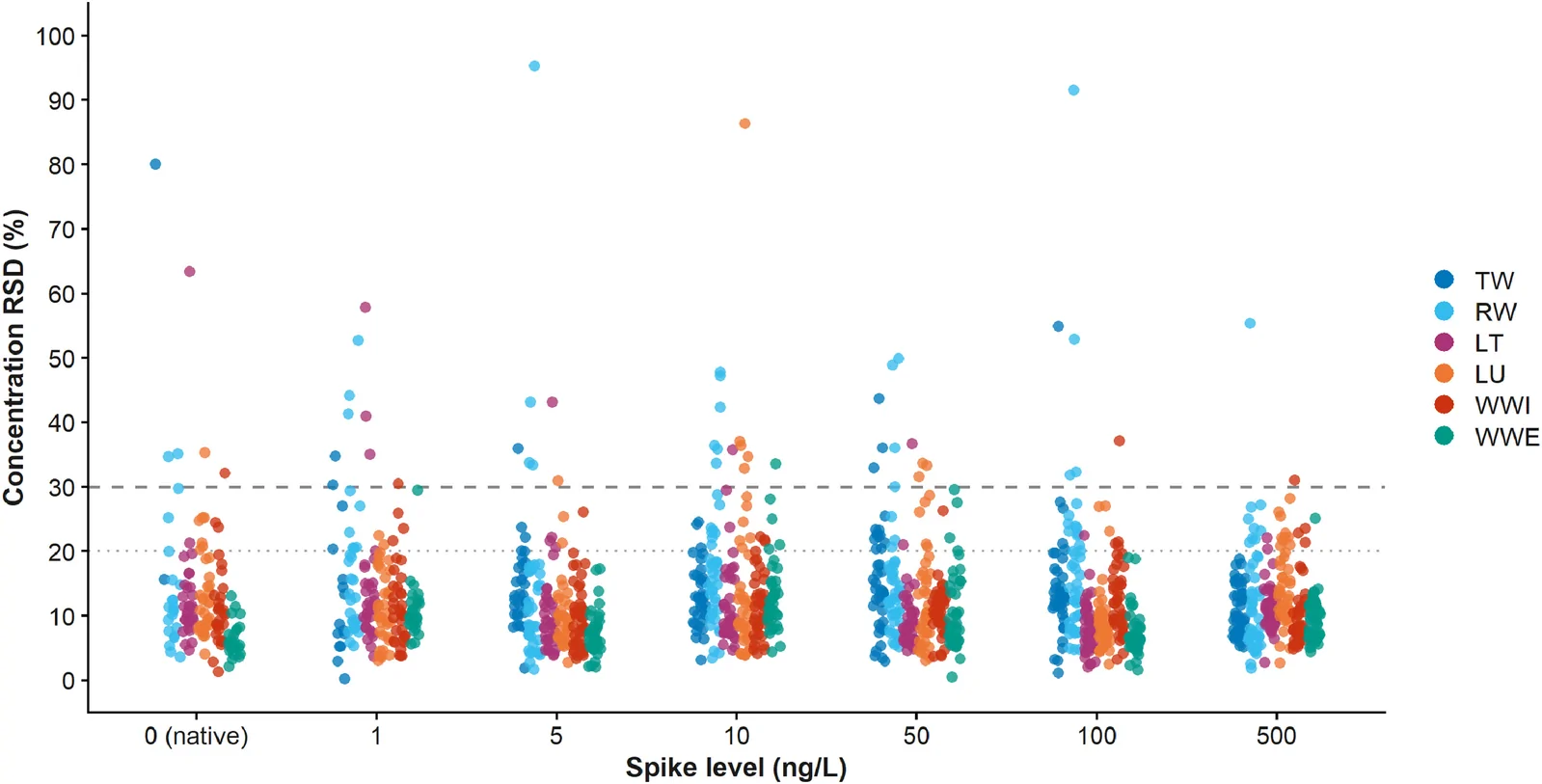
The precision of the analytical method in the different water matrices. The RSD is calculated from the concentration found at the 7 different spiking levels in six water matrices: tap water (TW), river water (RW), treated landfill leachate (LT), untreated landfill leachate (LU), wastewater influent (WWI), and wastewater effluent (WWE). The dashed line indicates 30% RSD; dotted line indicates the 20% RSD.

### Matrix specific LOQs, matrix effects and absolute recovery

The matrix specific LOQs, matrix effects and absolute recoveries for 46 compounds in six different water matrices are summarized in Fig. 3. LOQs were compound and matrix dependent, with most values ranging from 1 to 5 ng/L (Fig. 3a). For all matrices, the 75% quantile was below LOQ of 10.4 ng/L, except for WWI (<18.7 ng/L). Similar outliers were observed across matrices, with Nicotine, 2-Naphthalenesulfonic acid (2-NSA), Tris(1-chloro-2-propyl) phosphate (TCPP) and TBP, showing higher LOQ values (>50 ng/L).

**Fig. 3:**
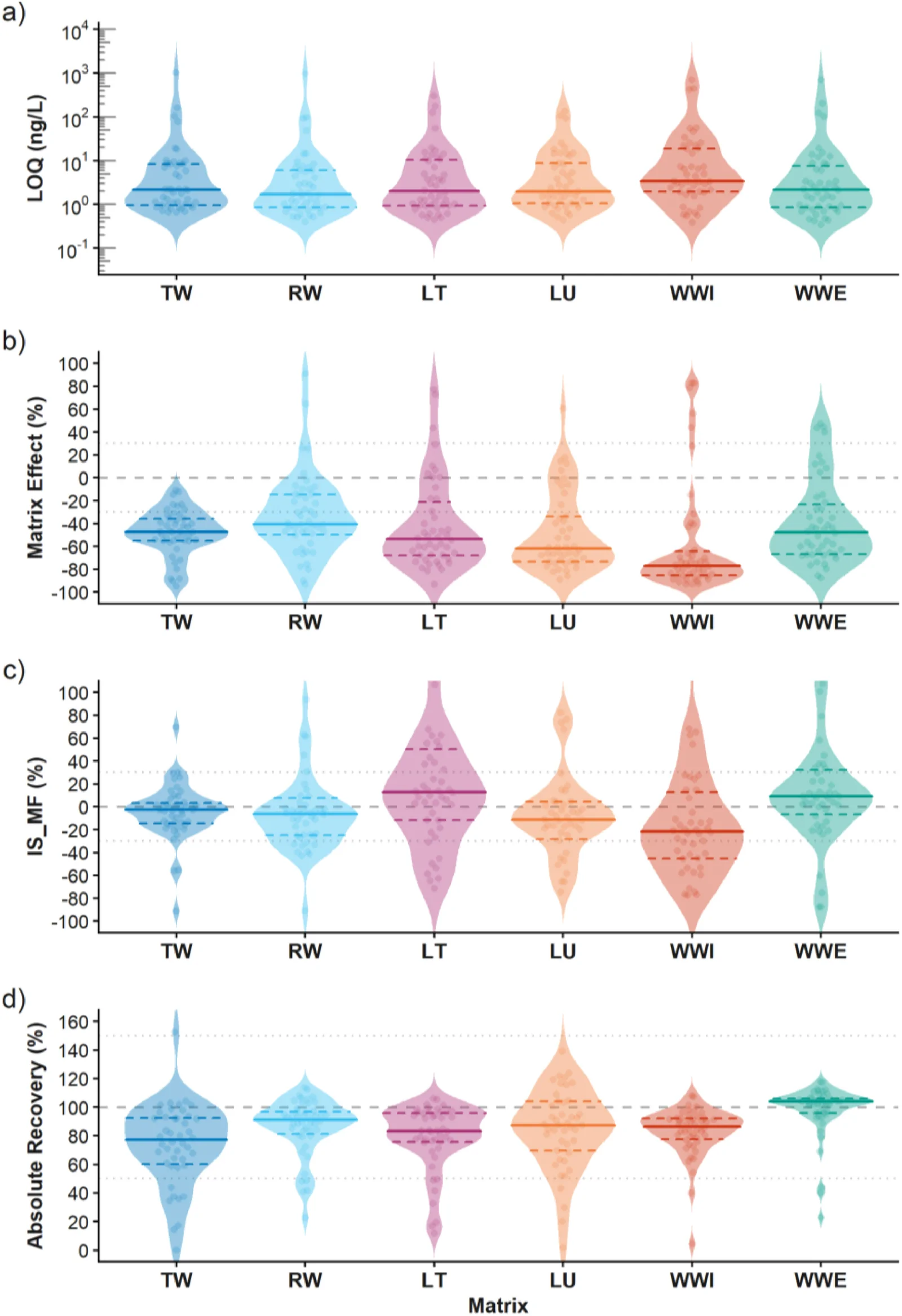
Violin plots of the six water matrices: tap water (TW), river water (RW), treated landfill leachate (LT), untreated landfill leachate (LU), wastewater influent (WWI), and wastewater effluent (WWE). With a) the Limit of Quantification (LOQ), b) the matrix effects without the presence of an internal standard, c) the matrix effects in the presence of an internal standard, d) the absolute recovery. (The LOQ axis is logarithmic; violin width represents the analyte distribution within each matrix, and the internal solid and dashed lines represents the median and first and third quartiles, respectively; horizontal reference lines indicate 0% and ±30% matrix effect in panels b and c and 100% and 50–150% recovery in panel d.

Matrix effects calculated without IS correction showed substantial ion suppression for most analysed compounds (Fig. 3b). Suppression was the most pronounced in WWI (median: -76.9%), compared with WWE (median: -47.7%) and downstream RW (median: -40.3%). Similarly, LU showed greater ion suppression (median: -61.8%) than LT (median: -53.6%). TW also showed ion suppression compared to MilliQ water (median: -47.2%), although variability between compounds was small, as indicated by the quantiles (± 5.7%).

Following IS’s correction, IS_MS for most compounds were within ±30% range, particularly in TW, RW and WWE (Fig. 3c). The IS_MF was observed in LT, LU and WWI, with both relative signal suppression and enhancement, depending on the compound and selected IS. Compounds for which isotopically labelled ISs were available generally showed the lowest IS_MS. In contrast, 1,3-Diphenylguanidine (DPG), 2-(4-(Diethylamino)-2-hydroxybenzoyl)benzoic acid (DHBA) and Nicotine, showed pronounced IS_MF with the current available ISs. Among the phosphates, TBP, TEP, Tris-(2-butoxyethyl) phosphate (TBEP) and TCPP showed relative signal enhancement (>30%). Whereas several industrial chemicals like, HMMM, BGA and Hexazinone, showed mainly relative signal suppression (<−30%).

For most compounds, absolute recoveries fell within the 50–150% interval (Fig. 3d). Broader recovery distributions were observed in TW, LT and LU. Several compounds, particularly the longer chain PFAS (PFUnDA, PFOS, PFDA and PFNA) and longer chain organo-phosphates, showed recoveries below 50%. Recovery values exceeding 150% were also observed with DPG in LU and TEP in TW. Overall, the validation results showed matrix-dependent variation in LOQs, matrix effects and absolute recoveries across the studied water matrices.

### Stability

The stability during sample storage was assessed in all six sample matrices for 46 native compounds by spiking native standard into bulk matrix and comparing the area ratio between native and IS with time 0 as reference. The stability for TW, RW, WWI, WWE matrices were evaluated after 1 W storage at +4°C (fridge) and 4 W at −20°C (freezer) (n=5), while LT and LU were evaluated at 1 W under both fridge and freezer condition as well as 2 and 4 W under freezer condition (n=2). A compound was considered stable when the mean stability fell within the range of 70%–130% of the reference value, consistent with common method validation criteria for trace organic analysis [11]. The stability result was presented in Table S5, and the 1 W and 4 W results were visualized as a violin plot in Fig. 4.

**Fig. 4:**
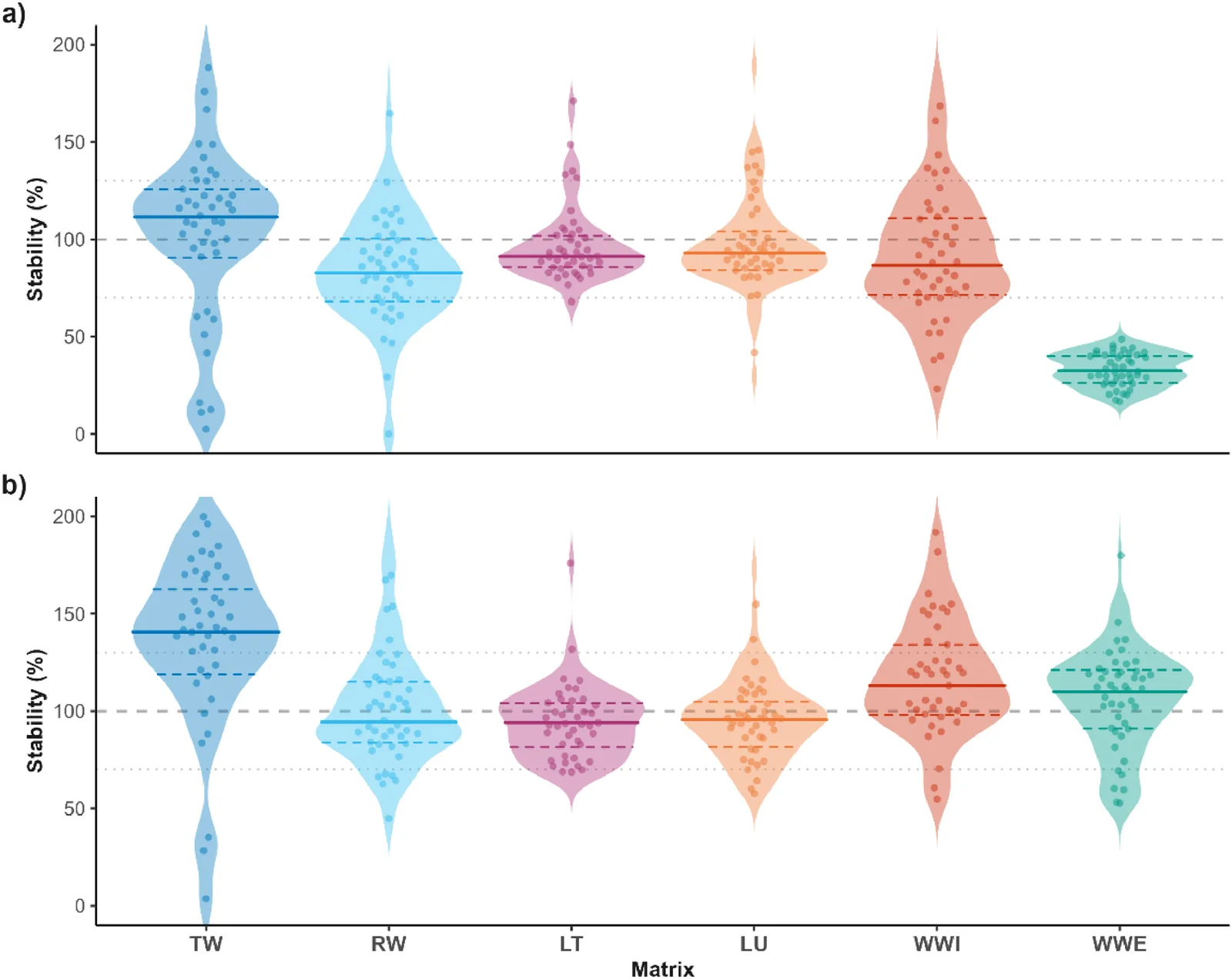
Violin plots of the stability of target compounds across different storage conditions across the different water matrices; tap water (TW), river water (RW), treated landfill leachate (LT), untreated landfill leachate (LU), wastewater influent (WWI), and wastewater effluent (WWE). With a) stored in fridge for one week, and b) stored in freezer for four weeks. (The violin width represents the analyte distribution within each matrix, and the internal solid and dashed lines represent the median and first and third quartiles, respectively; horizontal reference lines indicate 100% stability and 70% to 130% as acceptable stability range.).

Stability was strongly matrix dependent. LT and LU showed consistent behaviour, with both their first and third quartiles of median stability within the acceptable range for both timepoints, with 87%–88% compounds within the acceptable criteria. RW and WWI also demonstrated robust stability with 62%–76% of compounds within the acceptable range. In contrast, TW and WWE exhibited a rise in stability after storing in freezer for 4 W, compared with 1 W in fridge.

Short term storage in fridge (1 W, +4°C) was proven generally acceptable for most matrices. The median analyte stability for TW, RW, LT, LU, and WWI remained well within the 70% and 130% acceptance window. However, WWE exhibited a uniformly low stability profile under fridge conditions. After 1 W, the median stability of compounds in WWE dropped to approximately 40%, with the none of analytes passing the 70% threshold.

Freezer storage over 4 W effectively prevented the degradation observed in the WWE matrix, with the median stability for WWE around 110%. Consistent with the observation during short term storage, RW, LT, LU, and WWI remained within acceptable range. While freezer storage is highly recommended, especially for WWE, it is worth noting that TW exhibited the opposite pattern under this condition, with median stability exceeding 130% after 4 W.

The negative pattern for WWE (1 W) and positive pattern for TW (4 W) can possibly be explained by an analytical artifact. Because our stability evaluation relied on the native/IS peak area ratio relative to the time 0 reference, it is sensitive to changes of matrix effect. Unlike our routine quantification workflow, where native standard addition (MST) generates a matrix-specified slope, that can also reflect and compensate for matrix effect, the stability assessment was based on peak area ratio. Considering that not all target CECs are matched to their own isotopically labelled analog as IS, the compensation of matrix effect by non-analog IS could be less effective. During the storage period, the change of matrix can cause a bias on matrix effect, therefore influences on the stability assessment.

### Analysis of landfill leachate samples

Based on recovery result from method validation section, five phosphate esters (TBP, TEP, TiPP, TBEP, TCPP), 2-NSA, and Nicotine were excluded from the following analysis since they did not meet the performance criteria, narrowing the target list to from 46 to 39 CECs.

To demonstrate the applicability of the developed method in complex matrix, it was applied to LU and LT samples collected from five municipal landfills (Landfill A-E, detailed information about landfill can be found in [6]). The occurrence heat map was presented as Fig. 5, and the sum of CECs concentration was presented in Table S6.

**Fig. 5:**
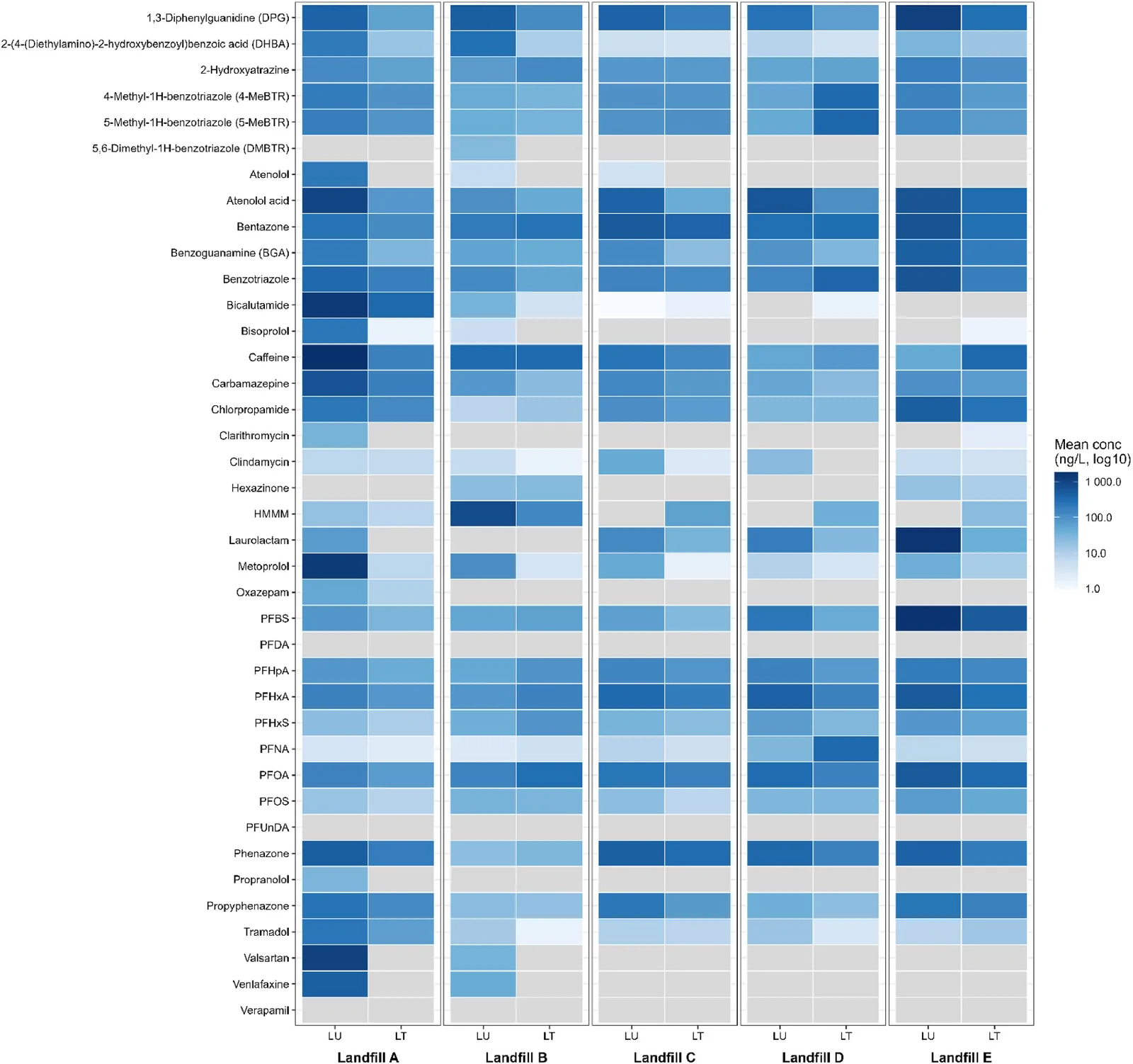
Occurrence heat map of 39 target compounds in untreated (LU) and treated leachate (LT) across five landfills (A-E). Cell colour indicates the mean concentrations (n=2), while grey cells indicate concentration below the matrix LOQ.

Among 39 target analytes, 36 of them were detected above LOQ in at least one site (only PFDA, PFUnDA, and Verapamil were never detected). Twenty-three (59%) CECs were quantified above LOQ in both LU and LT samples in all landfills, covering pharmaceuticals (*e.g.*, Atenolol acid, Carbamazepine), industrial chemicals (Benzotriazoles, DPG), pesticides (2-hydroxyatrazine), and PFAS (PFOA, PFHxA), underscoring the wide usage of these chemicals and limited efficiency of current treatment processes.

Measured concentrations spanned more than three orders of magnitude, from 0.91 ng/L (Bicalutamide in Landfill C’s LT) up to 1.84 μg/L (Caffeine, Landfill A’s LU). This wide range aligns with the vastly different usage of the target chemicals. In contrast, compounds such as PFHpA and 2-hydroxyatrazine were quantified in every landfill at comparatively consistent concentration, suggesting more spatially uniform sources.

The overall concentration profiles across the five reveal a highly variable distribution of CECs, heavily influenced by site-specific waste compositions. The sum of CECs concentration is presented in Table S6. The summed CEC concentration in LU ranged from 3.40 μg/L to 11.97 μg/L, with Landfill A roughly 1.2–3.5 times higher than the other four sites. As the heat map illustrated, Valsartan, Venlafaxine, Propranolol, Oxazepam were only detected in landfill A and/or B. Additionally, Atenolol, Bisoprolol, and Clarithromycin were also predominantly found in Landfill A.

These quantitative findings were broadly consistent with the qualitative non-target screening (NTS) result performed on samples from the same landfills [6]. The NTS study identified 76 contaminants of CEC across the five sites, including 43 CECs in LU and 34 remained detectable in LT. The widespread quantification of benzotriazoles, pharmaceuticals, pesticides, and seven PFAS supported the core spatial distribution pattern revealed by NTS study. Additionally, both studies indicated a more site-specific distribution of pharmaceuticals.

### Limitations

Not applicable.

## Ethics statements

The authors have read and follow the ethical requirements for publication in MethodsX. This work meets all ethic requirements. This work does not involve studies with animals and humans.

## CRediT authorship contribution statement

**Tsz Yung (Patrick) Wong:** Conceptualization, Data curation, Formal analysis, Investigation, Methodology, Writing – original draft. **Bent Speksnijder:** Conceptualization, Data curation, Formal analysis, Investigation, Methodology, Writing – original draft. **Gayani R. Kapukotuwa:** Formal analysis, Investigation, Writing – review & editing. **Oksana Golovko:** Conceptualization, Data curation, Investigation, Supervision, Resources, Writing – review & editing.

## Declaration of competing interest

The authors declare that they have no known competing financial interests or personal relationships that could have appeared to influence the work reported in this paper.

## Data Availability

Data will be made available on request.
